# Understanding differences in conception and abortion rates among under-20 year olds in Britain and France: Examining the contribution of social disadvantage

**DOI:** 10.1371/journal.pone.0186412

**Published:** 2017-10-16

**Authors:** Rachel H. Scott, Nathalie Bajos, Emma Slaymaker, Kaye Wellings, Catherine H. Mercer

**Affiliations:** 1 Department of Population Health, London School of Hygiene & Tropical Medicine, London, United Kingdom; 2 Department of Gender, Health and Sexuality, INSERM, Paris, France; 3 Department of Social and Environmental Health Research, London School of Hygiene & Tropical Medicine, London, United Kingdom; 4 Institute of Global Health, University College London, London, United Kingdom; University of Westminster, UNITED KINGDOM

## Abstract

**Objectives:**

Socioeconomic status has been shown to be associated with sexual activity, contraceptive-use, pregnancy and abortion among young people. Less is known about whether the strength of the association differs for each outcome, between men and women, or cross-nationally. We investigate this using contemporaneous national probability survey data from Britain and France.

**Methods:**

Data were analysed for 17–29 year-olds in Britain’s third National Survey of Sexual Attitudes and Lifestyles (Natsal-3, n = 5959) undertaken 2010–2012, and the 2010 French Fertility, Contraception and Sexual Dysfunction survey (FECOND, n = 3027). For each country, we estimated the gender-specific prevalence of sex before-16, contraceptive-use, conception before-20, and abortion in the event of conception, and used logistic regression to examine associations between two measures of socioeconomic status–educational-level and parental socioeconomic-group–and each outcome. We tested for interactions between socioeconomic characteristics and country, and socioeconomic characteristics and gender, for each outcome.

**Results:**

For each outcome, Britain and France differed with regard to prevalence but associations with socioeconomic characteristics were similar. Respondents of higher educational level, and, less consistently, with parents from higher socioeconomic-groups, were less likely to report sex before-16 (Britain, men: adjusted OR (aOR) 0.5, women: aOR 0.5; France, men: aOR 0.5, women: aOR 0.5), no contraception at first sex (Britain, men: aOR 0.4, women: aOR 0.6; France, men: aOR 0.4, women: aOR 0.4), pregnancy before-20 (Britain: aOR 0.3; France: aOR 0.1), and in Britain, a birth rather than an abortion in the event of conception (Britain: aOR 3.1). We found no strong evidence of variation in the magnitude of the associations with socioeconomic characteristics by country or gender.

**Conclusions:**

Population level differences in conception and abortion rates between the two countries may partly be driven by the larger proportion of the population that is disadvantaged in Britain. This research highlights the role intra-country comparisons can play in understanding young people’s sexual and reproductive behaviours.

## Introduction

Teenagers in Britain are more likely to become pregnant than those in France [1], and among those who do, young women in Britain are less likely to have an abortion [1]. Whilst it is well-established that under-20 conception and abortion are associated with socioeconomic status [2–6], this reading of the data can mask important nuances. Abortion is the result of a multistage pathway, which starts with first intercourse and contraceptive-use or non-use on this and later occasions, continues with the occurrence of unintended pregnancy, and ends with the decision to end the pregnancy and access abortion services [7,8]. Socioeconomic characteristics have been found to be associated with each stage in this pathway: in both countries women with lower educational attainment have been found to report earlier sexual debut [4,9], young people in Britain from more affluent backgrounds are more likely to use contraception and emergency contraception [4,10,11] and in France, condom-use at first sex is lower among women with less education [12]. In both countries young women from disadvantaged backgrounds are more likely to have an abortion if they become pregnant [13,14]. Socioeconomic disadvantage may influence sexual and reproductive health in several ways. As well as affecting young people’s expectations for their future and motivations to avoid pregnancy [5,15], advantaged young people have been found to be more knowledgeable about contraception and health services and better able to access them [16,17].

Little is known about whether the strength of the association between socioeconomic characteristics and each stage in the pathway (sexual activity, contraceptive-use, conception and abortion) varies cross-nationally. A five-country comparative study of socioeconomic disadvantage and young people’s sexual behaviour (using national-level aggregated survey data from the United States, Britain, France, Sweden and Canada) found that contraceptive-use at first intercourse differed according to socioeconomic indicators in Britain and the US, but not France [10], suggesting that the effects of socioeconomic characteristics may differ across country contexts. Furthermore, these associations may differ by gender. In Britain, parental social class was associated with age at first sex only among men [4], while in France, condom-use at first sex was found to be associated with educational-level only among women [12]. Gender differences in associations between socioeconomic characteristics and reported sexual and reproductive health outcomes may also vary cross-nationally, and may reflect differences in social pressures and expectations placed upon men and women [18]. Considering these differences by gender and cross-nationally may shed light on ways in which country-level differences in gender social structures might shape behaviours and thus risk of conception and abortion [18,19].

This paper presents a comparative analysis of the association of two specific socioeconomic characteristics–individual educational-level and parental socioeconomic group–with each stage of the pathway to abortion in Britain and France. This research complements the ecological analyses of Darroch, Singh et al. [10,20], which show socioeconomic gradients in sexual behaviour and reproductive health outcomes in five developed countries. We go further, using individual-level, rather than aggregated, data from two large, nationally-representative surveys with detailed and broadly comparable information on socioeconomic status and sexual and reproductive health outcomes. We also give a more contemporary picture by examining these relationships in a more current period. We chose these countries because whilst they are similar in many ways–being geographically close and sharing socio-demographic similarities–they have very different rates of teenage conception and abortion and differ in important ways that affect young people’s lives. In particular, the proportion of the population that is disadvantaged is greater in Britain: 21% of the British population has an income less than 60% of the median compared to 16% in France [21]. There is a wider gap between the incomes of the richest 20% and the poorest 20% in Britain compared to France [21]. Where income inequality is greater, so are social differences and so social stratification becomes more evident [22]. We can consider this in the context of young parenthood: young people may be more motivated to avoid pregnancy if they have a reasonable expectation of their opportunities for inclusion in society [5,23]. Where the gap between the richest and the poorest is wide, these expectations may become less reasonable among the more disadvantaged, and socioeconomic characteristics may have a stronger association with sexual and reproductive health outcomes than in a more egalitarian context. Even if the effect of socioeconomic characteristics were the same in both countries, greater inequality may lead to greater prevalence of sexual and reproductive health outcomes because of the relatively larger proportion of the population that is disadvantaged [24].

The aim of this paper is to better understand the role of two commonly-used measures of socioeconomic status at each stage of the pathway to abortion among young men and women in Britain and France, and their possible contribution to the variation in conception and abortion rates between the countries. We interpret the results with reference to known differences in the extent of country-level inequality.

## Methods

This paper draws on data from two nationally representative probability surveys, Britain’s third National Survey of Sexual Attitudes and Lifestyles (Natsal-3; total sample size 15,162) and France’s Fertility, Contraception and Sexual Dysfunction Survey (FECOND; total sample size 8,645). The Natsal study was approved by the Oxford Research Ethics Committee A [Ref: 10/H0604/27]. The FECOND study was approved by the relevant French government oversight agency (the Commission Nationale de l’Informatique et des Libertés) [n°909024]. Fieldwork for both surveys began in 2010. We focus on men and women aged 17–29 to present an accurate reflection of the contemporary situation, resulting initially in samples of 5,929 and 3,027 for Natsal-3 and FECOND respectively. Fieldwork for both surveys began in 2010. Natsal-3 used computer-assisted personal interviews (CAPI) with computer assisted self-administered interviews (CASI) for sensitive questions. FECOND was a telephone-survey, using landlines and mobiles. Natsal-3 used a multistage, clustered and stratified probability sampling strategy. In FECOND, two samples were independently selected to include a random sample of individuals who had a landline and a random sample of mobile phone users who did not, following a two stage random probability sampling process. Details of both methodologies are published elsewhere [25,26].

The response rate in the Natsal-3 survey was 57.7%. The data were weighted to adjust for unequal selection probabilities, and a non-response post-stratification weight corrected for differences in sex, age, and Government Office Region between the achieved sample and the 2011 census. In the FECOND survey, the total response rate was 50.2%. The data were weighted to adjust for unequal selection probabilities and post-stratification weights corrected for differences in sex, age, marital/cohabitation status, educational-level, professional situation, place of birth and dependent children between the achieved sample and the census. These surveys have the benefit of being conducted at the same point in time, and covered similar topics, facilitating comparability between the countries.

The key outcome variables are age at first heterosexual intercourse (dichotomised to before/after age 16), contraceptive-use at first sex, and among women only, reporting of a conception before age 20 and reporting of an abortion before age 20, among those who had conceived. Contraceptive-use at first intercourse was selected as an indicator of contraceptive-use over current use because we are interested in behaviours relating to conceptions before 20, and current use among older respondents may not accurately represent their contraceptive-use at younger ages. Contraception was defined as all medical methods of contraception and condoms.

The key independent variables in these analyses were respondent educational-level, an indicator of respondents’ individual social resources, and parental socioeconomic group, an indicator of respondents’ social origin. Respondent’s educational-level was defined as having completed some post-compulsory education or training *versus* having completed none, the latter being the reference category in logistic regression models. Sixteen year-olds were excluded from all analyses, as the school leaving age in both countries at the time of data collection was 16, so they may not have completed compulsory education at interview. Data on parental socioeconomic characteristics were collected differently in the two surveys; FECOND asked about parent’s educational-level, whereas Natsal-3 derived parent’s social class from parent’s occupation when the respondent was 14. We created a tiered variable with three tertiles in each country to capture relative socioeconomic group, on the grounds that educational-level is strongly associated with socioeconomic position [27]. Our data confirm this: 79% of participants aged 30–49 with a degree-level qualification in Natsal-3 and 74% in FECOND were in managerial and professional positions. In the British survey, we assigned parents who had never had a job or who were partly skilled or unskilled to the lower socioeconomic group, those in technical and skilled positions to the middle group, and those in professional and managerial occupations to the higher group. In the French survey, we assigned parents who had no qualifications to the lower group, those with baccalaureate or less to the middle group, and those with a degree-level qualification to the higher group. This variable measures relative, not absolute disadvantage. Non-response to questions on parent characteristics was relatively high–roughly ten percent–in both surveys (although missing data was less than 2% for all other variables [28]). Examination of item non-responders showed that on other characteristics they more closely resembled respondents from lower socioeconomic backgrounds. To avoid losing a large number of respondents from the analysis, and not bias the results towards respondents from higher socioeconomic groups, we created a fourth ‘not answered’ category, which we included in all analyses. We henceforth refer to this as parent relative socioeconomic group and those in the lower category are the reference category in logistic regression models.

### Analysis

We first described the two survey samples of men and women aged 17–29 years in terms of educational-level, parental relative socioeconomic group, and the outcomes on the pathway to abortion, and described differences between Britain and France and between men and women. Each analysis was restricted to respondents who had had the chance to experience the outcome of interest, e.g. analyses of contraceptive-use at first sex were run on respondents who reported ever having had sex. We used bivariate and multivariable logistic regression to examine these associations, adjusting for family structure at age 14/15 (whether the respondent lived with both natural parents at this age) as this was identified as a potential confounder in bivariate analyses. For all outcomes except reporting of sex before 16, we adjusted for age at first sex. To assess whether the strength of the association between socioeconomic characteristics and outcomes differed between men and women and between Britain and France, we tested for interactions of each of the two socioeconomic variables with sex and with country. All analyses were run on complete cases. Analyses were run using Stata Version 14, using the *svy* set of commands to account for clustering in the sampling.

## Results

Approximately two-thirds of women and men in Britain had completed any post-compulsory education (Table 1), while in France, 62% of men and 75% of women had completed any post-compulsory education. In both countries, less than 20% of men and women had parents in the lower socioeconomic group, roughly half in the middle group, and approximately one-quarter in the higher group.

**Table 1:** Characteristics of the sample in terms of socioeconomic characteristics and reporting of each outcome in the pathway to abortion, 17–29 year olds, Britain and France.

	Britain				France			
	Men		Women		Men		Women	
	n, N*	% (95%CI)	n, N*	% (95%CI)	n, N*	% (95%CI)	n, N*	% (95%CI)
**Total N aged 17–29**	2392		3327		1287		1740	
**Post-16 education or studying**	1770, 2355	68.6 (66.4–70.8)	1763, 3271	67.2 (65.3–69.0)	1170, 1286	61.5 (58.0–64.8)	1812, 1738	75.1 (72.5–77.6)
**Parent's socioeconomic group**								
Lower	1763, 2320	17.1 (15.5–18.8)	1751, 3217	18.9 (17.4–20.4)	1171, 1287	17.3 (14.7–20.3)	1815, 1740	18.6 (16.4–20.9)
Middle	1763, 2320	49.1 (46.7–51.5)	1751, 3217	49.8 (47.8–51.8)	1171, 1287	47.3 (44.1–50.6)	1815, 1740	44.8 (42.1–47.5)
Higher	1763, 2320	24.6 (22.5–26.8)	1751, 3217	21.3 (19.7–23.0)	1171, 1287	24.5 (22.0–27.2)	1815, 1740	24.6 (22.4–26.9)
Missing	1763, 2320	9.2 (8.0–10.7)	1751, 3217	10.0 (9.0–11.2)	1171, 1287	10.9 (9.0–13.1)	1815, 1740	12.1 (10.4–14.1)
**Had first het sex before age 16**	1757, 2308	26.6 (24.6–28.7)	1763, 3254	27.2 (25.5–29.0)	1149, 1266	27.0 (24.2–30.1)	1793, 1718	14.6 (12.8–16.6)
**No contraception at first sex**	1482, 1938	12.7 (11.1–14.6)	1520, 2854	12.0 (10.8–13.3)	979, 1077	6.9 (4.9–9.7)	1493, 1467	8.7 (6.9–10.9)
**Conception before age 20**	.	.	1162, 2200	25.6 (23.7–27.5)	.	.	1079, 1123	15.5 (13.0–18.3)
**Had an abortion before age 20, if conceived**	.	.	298, 667	32.2 (28.3–36.5)	.	.	169, 136	18.4 (12.4–26.6)

* n = weighted denominator, N = unweighted denominator. For condom at first sex, denominator is respondents who have ever had sex; for reporting of a conception before 20, denominator is women aged 20 and over who were sexually experienced by age 20; for reporting of an abortion before 20, denominator is women aged 20 and over, reporting a conception before age 20.

We found important and statistically significant differences between Britain and France in the proportions of men and women reporting outcomes at each stage in the pathway to abortion (Table 1). There was no difference between the two countries in the proportion of men reporting first sex before 16. However, 27% of women in Britain reported this compared to 15% in France. A smaller proportion of respondents in France reported using no contraception at first sex than in Britain. In both countries, these proportions were similar between men and women. A greater proportion of sexually-experienced women in Britain compared to France reported a conception before 20 (26% and 16% respectively). Finally, our data show that the proportion of women reporting an abortion in the event of a conception before 20 was higher in Britain (32%) than France (18%).

### First heterosexual sex before 16

In Britain, men and women with parents from a higher relative socioeconomic group were less likely to report sex before 16 than those with parents in a lower relative socioeconomic group (Table 2). In France, this was true only for men. The associations between parent relative socioeconomic group and reporting of sex before 16 were statistically significant in multivariate analyses only for men in Britain. In both countries, women and men with a higher educational-level were less likely to report sex before 16 in crude and multivariate analyses (Britain, men: aOR 0.5, women: aOR 0.4; France, men: aOR 0.5, women: aOR 0.5).

**Table 2:** Prevalence and odds of reporting first sex before 16 by parent socioeconomic group and individual level of education, 17–29 year olds, Britain and France.

	**Britain**											
	**Men**						**Women**					
	n, N*	**%(95% CI)**	**cOR(95%CI)**	**P-value**	**aOR and 95% CI**	**P-value**	n, N*	**%(95% CI)**	**cOR(95%CI)**	**P-value**	**aOR and 95% CI**	**P-value**
**Parent's socioeconomic group**												
Lower	289, 408	29.33 (24.79–34.33)	1	.	1	.	324, 618	30.50 (26.67–34.62)	1	.	1	.
Middle	843, 1092	27.68 (24.86–30.70)	0.92 (0.70–1.21)	0.558	1.09 (0.82–1.46)	0.54	858, 1568	26.84 (24.58–29.23)	0.84 (0.67–1.04)	0.101	1.10 (0.87–1.38)	0.419
Higher	428, 529	17.43 (14.23–21.17)	0.51 (0.37–0.70)	<0.001	0.70 (0.50–0.99)	0.044	369, 634	20.58 (17.44–24.12)	0.59 (0.45–0.77)	<0.001	0.87 (0.65–1.16)	0.338
Not answered	155, 217	36.54 (29.77–43.89)	1.39 (0.96–2.01)	0.084	1.14 (0.76–1.70)	0.537	169, 335	30.26 (25.20–35.86)	0.99 (0.72–1.35)	0.943	0.93 (0.67–1.29)	0.674
**Post-16 education or studying**												
None	522, 714	40.87 (36.89–44.98)	1	.	1	.	558, 1168	40.62 (37.51–43.82)	1	.	1	.
Some	1192, 1557	21.06 (18.85–23.46)	0.39 (0.31–0.48)	<0.001	0.45 (0.36–0.57)	<0.001	1170, 2030	21.31 (19.43–23.32)	0.40 (0.33–0.47)	<0.001	0.44 (0.37–0.53)	<0.001
	**France**											
	**Men**						**Women**					
	n, N*	**%(95% CI)**	**cOR(95%CI)**	**P-value**	**aOR and 95% CI**	**P-value**	n, N*	**%(95% CI)**	**cOR(95%CI)**	**P-value**	**aOR and 95% CI**	**P-value**
**Parent's socioeconomic group**												
Lower	196, 178	32.80 (25.08–41.59)	1	.	1	.	335, 291	15.01 (10.96–20.21)	1	.	1	.
Middle	552, 622	26.53 (22.46–31.03)	0.74 (0.48–1.14)	0.176	0.88 (0.55–1.40)	0.584	801, 799	14.65 (12.08–17.65)	0.97 (0.64–1.49)	0.895	1.08 (0.70–1.66)	0.742
Higher	284, 352	22.49 (17.68–28.16)	0.59 (0.37–0.96)	0.035	0.88 (0.52–1.50)	0.637	442, 452	12.92 (9.83–16.80)	0.84 (0.52–1.35)	0.472	1.07 (0.65–1.76)	0.781
Not answered	117, 114	30.71 (22.28–40.67)	0.91 (0.51–1.62)	0.743	0.79 (0.43–1.46)	0.456	214, 176	17.23 (11.96–24.19)	1.18 (0.67–2.06)	0.564	0.87 (0.48–1.57)	0.643
**Post-16 education or studying**												
None	441, 348	36.16 (30.53–42.18)	1	.	1	.	438, 323	22.50 (17.98–27.78)	1	.	1	.
Some	708, 917	21.35 (18.38–24.66)	0.48 (0.35–0.66)	<0.001	0.52 (0.37–0.73)	<0.001	1352, 1393	12.07 (10.29–14.11)	0.47 (0.34–0.66)	<0.001	0.50 (0.34–0.71)	<0.001

*n = weighted denominator, N = unweighted denominator. aOR adjusted for family structure at age 14/15

### Contraception at first sex

In Britain and France, women with parents from a middle or higher relative socioeconomic group, and men with parents from a higher socioeconomic group were less likely to report using no contraception at first sex (Table 3). In multivariate analyses, the association between parent relative socioeconomic group and reporting of no contraceptive-use at first sex was significant among women in Britain and France, and men in France. In both countries, men and women with a higher educational-level were less likely to report no contraception at first sex in crude and multivariate analyses (Britain, men: aOR 0.4, women: aOR 0.6; France, men: aOR 0.4, women: aOR 0.3).

**Table 3:** Prevalence and odds of reporting no contraceptive use at first sex by parent socioeconomic group and individual level of education, 17–29 year olds, Britain and France.

	**Britain**											
	**Men**						**Women**					
	n, N*	**%(95% CI)**	**cOR(95%CI)**	**P-value**	**aOR and 95% CI**	**P-value**	n, N*	**%(95% CI)**	**cOR(95%CI)**	**P-value**	**aOR and 95% CI**	**P-value**
**Parent's socioeconomic group**												
Lower	234, 332	15.92 (11.92–20.94)	1	.	1	.	279, 544	16.16 (13.09–19.79)	1	.	1	.
Middle	717, 927	12.37 (10.09–15.08)	0.75 (0.50–1.11)	0.146	0.77 (0.51–1.16)	0.217	747, 1396	8.93 (7.47–10.65)	0.51 (0.37–0.70)	<0.001	0.53 (0.39–0.74)	<0.001
Higher	366, 446	9.42 (6.78–12.95)	0.55 (0.34–0.89)	0.014	0.75 (0.45–1.23)	0.248	315, 538	10.09 (7.49–13.48)	0.58 (0.39–0.88)	0.01	0.64 (0.42–0.98)	0.039
Not answered	129, 181	14.02 (9.13–20.94)	0.86 (0.47–1.56)	0.623	0.78 (0.42–1.45)	0.43	138, 285	20.34 (15.70–25.93)	1.32 (0.89–1.96)	0.161	1.08 (0.71–1.65)	0.716
**Post-16 education or studying**												
None	471, 644	19.87 (16.40–23.86)	1	.	1	.	518, 1093	15.21 (13.07–17.63)	1	.	1	.
Some	980, 1265	8.76 (7.13–10.71)	0.39 (0.28–0.53)	<0.001	0.43 (0.30–0.61)	<0.001	974, 1713	9.44 (7.95–11.17)	0.58 (0.45–0.75)	<0.001	0.62 (0.47–0.81)	0.001
	**France**											
	**Men**						**Women**					
	n, N*	**%(95% CI)**	**cOR(95%CI)**	**P-value**	**aOR and 95% CI**	**P-value**	n, N*	**%(95% CI)**	**cOR(95%CI)**	**P-value**	**aOR and 95% CI**	**P-value**
**Parent's socioeconomic group**												
Lower	164, 151	13.93 (6.71–26.69)	1	.	1	.	277, 244	17.84 (11.95–25.79)	1	.	1	.
Middle	478, 530	6.42 (4.00–10.16)	0.42 (0.16–1.10)	0.077	0.49 (0.19–1.26)	0.139	673, 693	4.72 (3.11–7.10)	0.23 (0.12–0.43)	<0.001	0.30 (0.16–0.56)	<0.001
Higher	246, 305	1.36 (0.60–3.06)	0.09 (0.03–0.27)	<0.001	0.15 (0.05–0.46)	0.001	368, 385	6.44 (3.85–10.58)	0.32 (0.15–0.65)	0.002	0.53 (0.26–1.06)	0.074
Not answered	92, 91	11.63 (6.01–21.29)	0.81 (0.27–2.40)	0.708	0.78 (0.27–2.28)	0.646	175, 145	14.43 (9.24–21.83)	0.78 (0.39–1.55)	0.472	0.61 (0.30–1.25)	0.177
**Post-16 education or studying**												
None	386, 312	12.35 (7.83–18.93)	1	.	1	.	394, 289	16.96 (12.19–23.10)	1	.	1	.
Some	593, 765	3.35 (2.21–5.04)	0.25 (0.13–0.48)	<0.001	0.39 (0.19–0.80)	0.010	1097, 1176	5.77 (4.28–7.73)	0.30 (0.18–0.49)	<0.001	0.34 (0.20–0.58)	<0.001

*n = weighted denominator, N = unweighted denominator. Denominator restricted to respondents who had ever had sex. aOR adjusted for family structure at age 14/15 and age at first sex

### Conception before 20

In both countries, women with parents from middle and higher relative socioeconomic groups were less likely to report conceiving before 20 (Table 4). In multivariate analyses this association remained significant only in Britain. Women with a higher educational-level were less likely to report conceiving before 20 in both countries in crude and multivariate analyses (Britain: aOR 0.3; France: aOR 0.1).

**Table 4:** Prevalence and odds of reporting a conception before age 20 by parent socioeconomic group and individual level of education, 17–29 year olds, Britain and France.

	**Britain**					
	**Women**					
	n, N*	**%(95% CI)**	**cOR(95%CI)**	**P-value**	**aOR and 95% CI**	**P-value**
**Parent's socioeconomic group**						
Lower	228, 442	34.42 (29.83–39.31)	1	.	1	.
Middle	563, 1057	21.79 (19.30–24.51)	0.53 (0.41–0.69)	<0.001	0.73 (0.55–0.98)	0.035
Higher	232, 399	13.96 (11.07–17.45)	0.31 (0.22–0.43)	<0.001	0.58 (0.40–0.84)	0.004
Not answered	107, 231	40.76 (34.12–47.75)	1.31 (0.93–1.86)	0.127	1.10 (0.72–1.66)	0.667
**Post-16 education or studying**						
None	442, 943	44.28 (40.88–47.74)	1	.	1	.
Some	700, 1220	13.84 (11.94–15.99)	0.20 (0.16–0.25)	<0.001	0.27 (0.21–0.35)	<0.001
	**France**					
	**Women**					
	n, N*	**%(95% CI)**	**cOR(95%CI)**	**P-value**	**aOR and 95% CI**	**P-value**
**Parent's socioeconomic group**						
Lower	196, 188	21.67 (15.24–29.85)	1	.	1	.
Middle	485, 530	13.01 (9.84–17.03)	0.54 (0.32–0.92)	0.024	0.60 (0.33–1.06)	0.079
Higher	269, 297	6.65 (3.66–11.78)	0.26 (0.12–0.55)	<0.001	0.61 (0.27–1.41)	0.25
Not answered	129, 108	33.61 (24.28–44.42)	1.83 (0.98–3.43)	0.059	0.96 (0.47–1.97)	0.912
**Post-16 education or studying**						
None	330, 249	38.03 (31.51–45.01)	1	.	1	.
Some	746, 872	5.33 (3.89–7.27)	0.09 (0.06–0.14)	<0.001	0.12 (0.07–0.20)	<0.001

*n = weighted denominator, N = unweighted denominator. Denominator restricted to women aged 20 and over, sexually experienced by age 20. aOR adjusted for family structure at age 14/15 and age at first sex.

### Abortion before 20, where conception occurred

In Britain, women with parents from a higher relative socioeconomic group were more likely to report an abortion in the event of conceiving before age 20 than those with parents from a lower socioeconomic group (Table 5). In multivariate analyses this association was not statistically significant. In Britain, women with a higher educational-level were more likely to report an abortion in the event of conceiving before 20 in crude and multivariate analyses (Britain: aOR 3.1).

**Table 5:** Prevalence and odds of reporting an abortion before age 20, among women who conceived before age 20, by parent socioeconomic group and individual level of education, 17–29 year olds, Britain and France.

	**Britain**					
	**Women**					
	n, N*	**%(95% CI)**	**cOR(95%CI)**	**P-value**	**aOR and 95% CI**	**P-value**
**Parent's socioeconomic group**						
Lower	80, 176	31.85 (24.61–40.09)	1	.	1	.
Middle	122, 269	33.44 (27.27–40.24)	1.08 (0.69–1.68)	0.75	0.91 (0.56–1.50)	0.718
Higher	33, 69	51.10 (38.23–63.84)	2.24 (1.21–4.15)	0.011	1.57 (0.83–3.00)	0.167
Not answered	44, 104	16.88 (9.67–27.83)	0.43 (0.21–0.91)	0.027	0.37 (0.18–0.79)	0.01
**Post-16 education or studying**						
None	196, 457	23.44 (19.51–27.89)	1	.	1	.
Some	98, 198	50.68 (42.67–58.65)	3.36 (2.26–4.99)	<0.001	3.14 (2.05–4.80)	<0.001
	**France**					
	**Women**					
	n, N*	**%(95% CI)**	**cOR(95%CI)**	**P-value**	**aOR and 95% CI**	**P-value**
**Parent's socioeconomic group**						
Lower	43, 33	16.90 (7.21–34.71)	1	.	1	.
Middle	63, 53	15.79 (8.05–28.65)	0.92 (0.27–3.14)	0.897	0.75 (0.22–2.54)	0.645
Higher	18, 16	17.81 (4.83–48.06)	1.07 (0.19–6.08)	0.943	0.93 (0.13–6.99)	0.948
Not answered	45, 34	23.91 (11.48–43.21)	1.55 (0.42–5.71)	0.514	1.18 (0.31–4.49)	0.811
**Post-16 education or studying**						
None	127, 88	17.63 (10.74–27.58)	1	.	1	.
Some	40, 47	18.20 (8.84–33.79)	1.04 (0.38–2.86)	0.94	1.25 (0.38–4.16)	0.715

*n = weighted denominator, N = unweighted denominator. Denominator restricted to women aged 20 and over, reporting a conception before age 20. aOR adjusted for family structure at age 14/15 and age at first sex.

**Fig 1:**
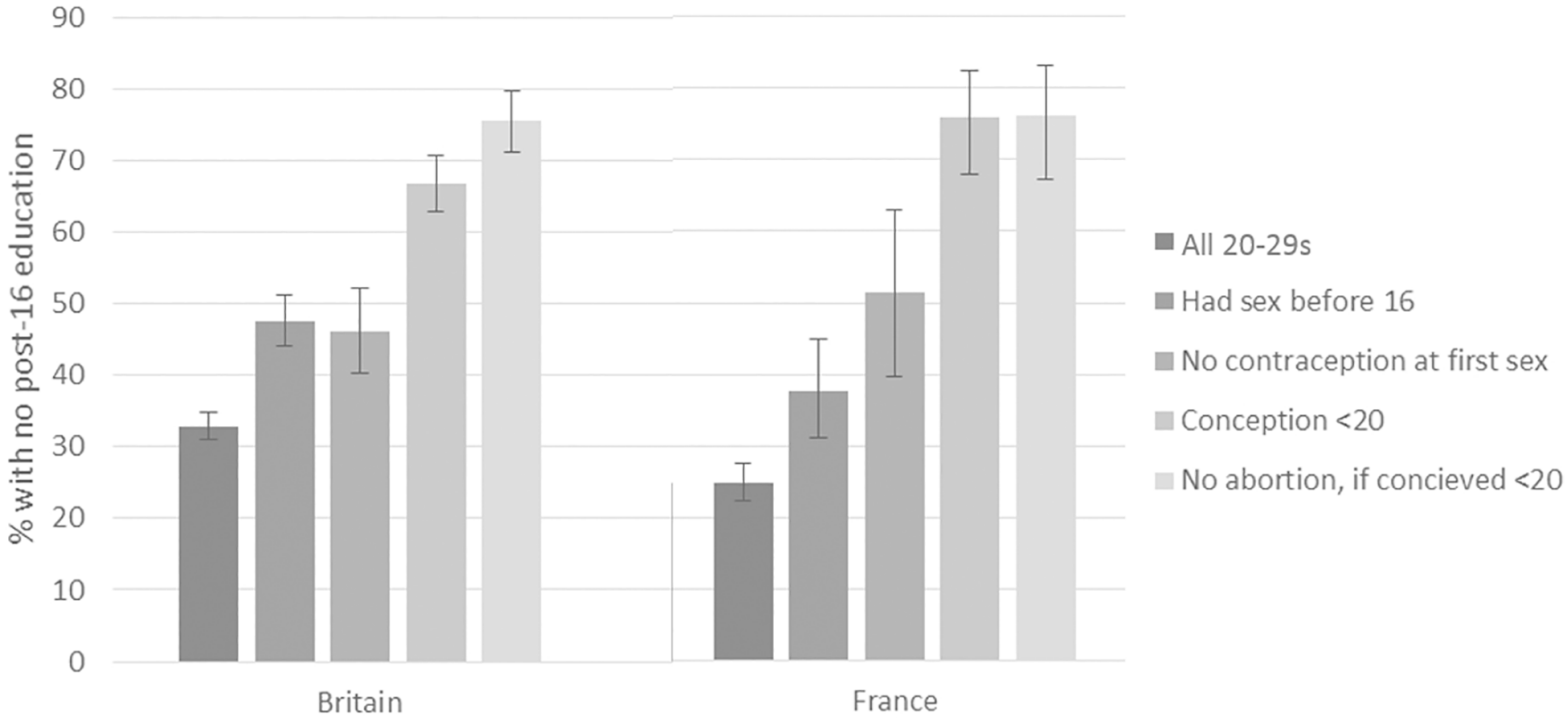
Distribution of level of education among women reporting successive sexual health outcomes, 20-29s, Britain and France. For contraception at first sex, denominator is women who have ever had sex; for conceptions before 20, denominator is women aged 20 and over who were sexually experienced by age 20; for no abortion if conceived before 20, denominator is women aged 20 or over who reported a conception before age 20.

With each sequential stage in the pathway to abortion, the composition of the sample reporting that outcome changes, reflecting the associations described above between socioeconomic characteristics and sexual health behaviours and outcomes. Fig 1 shows the percentage with no post-compulsory education among all women aged 20–29, those reporting first sex before 16, not using contraception at first sex, reporting a conception before 20 (among those sexually experienced), and not reporting an abortion (among those who conceived). This illustrates how at each successive outcome, the composition of the sample reporting that outcome becomes increasingly disadvantaged (in this case less educated) compared to the whole population and the preceding stage.

In Britain and France, testing for interactions found no evidence of differences between men and women in the strength of the association between socioeconomic characteristics and sex before 16 or contraceptive-use at first sex. The strength of the association between socioeconomic characteristics and each outcome was also remarkably similar between countries. There was no evidence of between-country differences in the strength of the association between parent relative socioeconomic group and any of the outcomes, or in the association between respondent educational-level and sex before 16 or contraceptive-use at first sex. The association between respondent educational-level and conception before 20 among women was stronger in France than in Britain (interaction term 0.49, p<0.01), and the association between respondent educational-level and abortion in the event of conception was statistically significant in Britain but not France (interaction term 0.28, p = 0.02).

## Discussion

To our knowledge, this is the first paper to consider associations with socioeconomic characteristics at each stage in the pathway to abortion, and to examine whether the associations observed differ between men and women and cross-nationally. The findings reveal both notable differences and remarkable similarities between Britain and France in terms of sexual behaviour, contraceptive-use and reproductive events reported by young men and women. At each stage of the process leading to abortion, Britain and France differed in the proportion reporting each outcome, yet showed a similar association with socioeconomic characteristics. In both countries, and consistent with previous literature [4,6,7,9,13,14], there was a cumulative pattern with socioeconomic characteristics in the pathway to abortion whereby respondents with less education (and, less consistently, with parents from a lower socioeconomic group) were more likely to report first sex before 16, to not use contraception at first sex, and among women, to report a pregnancy before 20 and to take it to term if they did.

We found evidence of interactions only in the associations between educational-level and conception and recourse to abortion before 20 among women. The association between educational-level and conception before 20 was stronger in France than Britain, while the association between educational-level and recourse to abortion before 20 was strong in Britain but not statistically significant in France, suggesting that abortion decision-making may be more socially-stratified in Britain. These findings should be interpreted with caution. Since we found no evidence for between-country differences in the associations between socioeconomic characteristics and sexual activity and contraceptive-use, the ‘antecedents’ of conception and abortion, between-country differences in the associations between educational-level and conception and abortion outcomes might result from differential misclassification that results from underreporting of abortion in both surveys [29], combined with small numbers reporting these outcomes. With a larger sample, the association between educational-level and recourse to abortion in France may have reached statistical significance.

Britain and France both have comprehensive health and social welfare systems. However, Britain is a society that is more marked by socioeconomic inequalities than France [21,20]. As has been demonstrated previously in both countries [4,6,7,9,13,14], sexual and reproductive health outcomes were associated with socioeconomic characteristics, particularly educational-level. We found no strong evidence for our first hypothesis that in a context of greater socioeconomic inequality (Britain) the association between socioeconomic status and sexual and reproductive health outcomes would be stronger. However, our results suggest that population-level differences in prevalence of sexual health outcomes may be partly driven by country-level differences in inequality and degree of socioeconomic disadvantage. There are striking differences in population-level conception and abortion rates among under-20s obtained from national statistics between Britain and France, yet the associations between socioeconomic characteristics and the sexual and reproductive health outcomes studied in this paper are similar in both countries. The greater levels of social inequality in Britain compared to France means that a greater proportion of young people in Britain are disadvantaged [21,30]. The differences in conception and abortion rates between the two countries may be due in part to differences in the proportion of individuals that are more ‘at risk’ of experiencing these outcomes. Whilst this hypothesis as an explanation for cross-national differences in teenage conception rates has been proposed before [10,24], to our knowledge, it has not been examined empirically. Our use of high quality, nationally-representative, individual-level data are further strengths of this paper.

Other social-contextual factors may also be important, particularly those that affect young people or relate to their motivations to avoid pregnancy. Young parenthood can be an alternative means of attaining an adult social status among those to whom traditional routes, through education and employment, seem less attainable [5]. Van de Velde [31] argues that the transition to adulthood is experienced differently in Britain and France, with a rapid transition to independence in Britain, whilst in France youth is considered a time of investment, with a focus on education. In France, therefore, becoming a parent early goes very much against social norms, whilst in Britain it can be compatible with a transition to adulthood that encourages early independence. Motivations to avoid or delay parenthood translate into sexual behaviours and contraceptive-use; young people for whom education is important may prioritise this over romantic relationships and sexual initiation [32], and may be more committed to using contraception consistently and effectively.

There was no evidence that the associations between socioeconomic characteristics and sexual activity or contraceptive-use at first sex varied between men and women in either country, or of gender differences in the proportion reporting no contraception at first sex. However, in France but not Britain, fewer women than men reported first sex before 16. In France, it appears that although social disadvantage is important in shaping the timing of first sex, shown in the greater proportion of respondents with a lower educational-level reporting sex before 16, it is also perhaps a more strongly gendered event than in Britain. Previous research has shown larger age gaps between partners at first sex in France than in Britain [29], which may also reflect more strongly gendered social norms. These specific gender differences present in France but not Britain highlight the important role of country context; these more gendered elements of first sex in France may reflect a more gender unequal social structure [18,29].

In both countries, the respondent’s social origin (parent relative socioeconomic group) was less consistently associated with each outcome than the respondent’s social resources (respondent educational-level). This may reflect the declining social control of families in European societies, a result of which is that regulation of young people’s sexuality is increasingly governed more by peers than by parents [33]. It is therefore not surprising to find a less-marked association with social origin than individual social resources. Leaving school at 16 reflects a different expected trajectory, different peers and a different social milieu compared to continuing education; this translates into differences in behaviours.

Between-country differences in health care systems may affect young people’s contraceptive-use. In Britain, contraception is available free of charge and without age-related restriction, from multiple sources [34]. In France, contraception is partially reimbursed by health insurance, but under-18s are under parental health cover so can access it anonymously and for free only through family planning clinics which are unevenly distributed across the country [35]. In this analysis, greater access to contraception in Britain did not translate into smaller socioeconomic disparities in contraceptive-use among young people relative to France. This, and the finding that more young people in France use contraception at first sex despite greater availability in Britain, further supports the contention that social-contextual factors affecting young people’s motivations to avoid pregnancy and parenthood shape sexual and reproductive health outcomes.

### Strengths and limitations

Key strengths of this study include being able to analyse broadly comparable, individual-level data from national probability surveys from two countries to contrast how two commonly-used indicators of socioeconomic status relate to a number of sexual and reproductive health outcomes experienced on the pathway to abortion. Although data on parental characteristics were collected differently in the two surveys, which may capture different elements of socioeconomic position in Britain and France, we were able to create a *relative* measure by constructing a tiered variable.

Contraceptive-use at first sex as an indicator of contraceptive-use at a young age is a crude indicator that does not capture all the nuances of contraceptive-use, for example method choice or consistency of use, both of which may change over time [36](. That said, contraceptive-use at first sex is an indicator of contraceptive-use for all sexually experienced respondents, not just those currently sexually active, and previous research has shown that contraceptive-use at first sex is a strong predictor of current contraceptive-use among young people [37](.

As this is a cross-sectional study, we can neither assume causality nor rule out reverse causality. Educational-level might influence an individual’s timing of sexual debut or their likelihood of becoming pregnant or having an abortion before age 20 [38]. However, it is also possible that a conception or abortion before age 20, or early sexual debut, might influence an individual’s likelihood of continuing education [39–41]. This might also differ between the two countries. If young women in Britain are less likely to continue with their education after an abortion, but their French counterparts are not, this may partly explain our finding that level of education was associated with recourse to abortion in Britain but not in France. Further research using longitudinal data would enhance our understanding of the direction of these associations.

Although in previous Natsal surveys reporting of abortions has been high [42](, abortions are underreported in both surveys [29]. This affects the accuracy of both the conception and the abortion figures. Of greater concern for this study is that underreporting is not random. In the US National Survey of Family Growth, reporting varied by age, income and educational-level [43]. If reporting is biased towards women in higher socioeconomic groups, our study may underestimate the associations between socioeconomic status and conception and abortion. Another consequence of underreporting is that the numbers reporting abortion in both surveys are low, particularly in France where abortion is rarer and the sample size smaller, making interpretation of results more difficult due to lack of statistical power. However, detailed data on sexual behaviour and contraception, factors ‘upstream’ from conception and abortion, can inform the interpretation of our findings on conception and abortion.

The associations observed in this study may also have been biased by differential reporting of sexual debut and contraceptive-use among people of different socioeconomic status or between men and women. This would undermine the between-country comparisons if this bias differed between Natsal-3 and FECOND. For example, if in France but not in Britain, strong social norms around male and female sexuality led to men over reporting and women underreporting sex before age 16, the reported differences between men and women in age at first sex observed in France but not in Britain might reflect social influences on reporting rather than a true difference in behaviours.

## Conclusions

Conception and abortion rates observed in national statistics are markedly higher in Britain than in France, yet in both countries there is a strong and similar association between socioeconomic characteristics and outcomes at each stage in the pathway to abortion. Britain is a society marked by higher levels of disadvantage compared to France [21,30] and this analysis lends empirical support to the hypothesis that population-level differences in conception and abortion rates may be partly attributable to a greater proportion of the population that is disadvantaged and more ‘at risk’ [10,24]. Differences in broader social-contextual factors may also influence young people’s behaviours and decision-making. Differences in the way in which the transition to adulthood is experienced in the two countries may mean that motivations to avoid or delay pregnancy may be lesser in Britain. In addition, gender differences in the timing of sexual debut in France but not Britain suggest that more strongly gendered social norms in France might also shape sexual behaviours. Future research should explore the mechanisms through which social disadvantage affects sexual behaviour and contraceptive use, paying particular attention to motivations to avoid pregnancy.

## Supporting information

S1 Full Methods (DOCX)
