# Supplementary material for: Understanding differences in conception and abortion rates among under-20 year olds in Britain and France: Examining the contribution of social disadvantage
Source: PLoS One. 2017 Oct 16;12(10):e0186412. doi: 10.1371/journal.pone.0186412 (PMC5643109; doi:10.1371/journal.pone.0186412)
Supplement: S1 Full Methods — (DOCX) [file pone.0186412.s001.docx]

**S1. Full methods**

This paper draws on data from two nationally representative probability surveys, Britain’s third National Survey of Sexual Attitudes and Lifestyles (Natsal-3; total sample size 15,162) and France’s Fertility, Contraception and Sexual Dysfunction Survey (FECOND; total sample size 8,645). We focus on men and women aged 17-29 to present an accurate reflection of the contemporary situation, resulting in samples of 5,929 and 3,027 for Natsal-3 and FECOND respectively. Fieldwork for both surveys began in 2010. Natsal-3 used computer-assisted personal interviews (CAPI) with a computer assisted self-administered interview (CASI) for sensitive questions. FECOND was a telephone-survey, using landlines and mobiles. Natsal-3 used a multistage, clustered and stratified probability sampling strategy; within each primary sampling unit (postcode sectors), addresses were randomly selected. In FECOND, two samples were independently selected to include a random sample of individuals who had a landline and a random sample of mobile phone users who did not have a landline, following a two stage random probability sampling process. An initial probability sample of households or mobile phones was selected using random digit dialling, and one eligible individual per household or mobile phone was randomly selected for participation. Details of both methodologies are published elsewhere [1,2].

The response rate in the Natsal-3 survey was 57.7%, after taking into account non-eligible subjects. The Natsal-3 data were weighted to adjust for the unequal probabilities of selection in terms of age and number of eligible adults in the household. After weighting, the sample was broadly representative of the British population in the 2011 census. Men and London residents were slightly underrepresented, so a non-response post-stratification weight was applied to correct for differences in sex, age, and Government Office Region between the achieved sample and the 2011 census.

The response rate in the FECOND survey was 54.1% for the landline sample and 37.6% for the mobile phone sample, after taking into account non-eligible subjects. The total response rate was 50.2%. The FECOND data were weighted to adjust for the unequal probabilities of selection in the sample in terms of age, sex and landline or mobile interview. After weighting, some groups were underrepresented in comparison to the French census collected continuously from 2005-2009, particularly individuals born outside of France, and with no qualifications. Post-stratification weights corrected for differences in sex, age, marital/cohabitation status, educational-level, professional situation, place of birth and dependent children between the achieved sample and the census.

These surveys have the benefit of being conducted at the same point in time, and covered similar topics, facilitating comparability between the countries. Both gathered detailed information on sexual behaviour, as well as information on the socioeconomic characteristics of respondents, including their educational-level at the time of interview and socioeconomic characteristics of their parents.

The key outcome variables relevant to the questions addressed in this paper were age at first heterosexual intercourse (dichotomised to before/after age 16), contraceptive-use at first sex, and among women only, reporting of a conception before 20 and reporting of an abortion before 20, among those who had conceived. Use of a reliable method of contraception at first intercourse was selected as an indicator of contraceptive-use in preference to current method of contraception because we are interested in behaviours relating to conceptions before 20, and current contraceptive-use among older respondents may not be an accurate representation of their contraceptive-use at younger ages. Reliable methods include all medical methods of contraception and condoms, and exclude withdrawal, periodic abstinence and no method.

Abortions are known to be underreported in surveys [3]. This affects the accuracy of both the conception and the abortion figures, analyses of which must be interpreted with caution. When comparing the estimated birth and abortion rates to under-20s as reported in the surveys to national statistics, abortions are underreported in both surveys there is no evidence that the extent of underreporting of abortions varies between the surveys [4]. Miscarriages are not included in these analyses. Men’s reports of conceptions and abortions occurring to their partners were not collected in both surveys.

The key independent variables in these analyses were respondent educational-level, an indicator of the respondent’s individual social resources, and their parent’s socioeconomic group, an indicator of the respondent’s social origin. Respondent’s educational progression, derived from information on the highest qualification achieved and current educational activity, was defined as having completed some post-compulsory education or training *versus* having completed none (the minimum school leaving age in Britain and France at the time of data collection was 16). We henceforth refer to this as respondent educational-level. Sixteen year olds were excluded from all analyses as they may not have completed compulsory education at the time of interview. Data on parental socioeconomic characteristics were collected differently in the two surveys; FECOND asked about parent’s educational-level, whereas Natsal-3 derived parent’s social class from questions about parent’s occupation and responsibility when the respondent was 14. We created a scale of relative socioeconomic group with three levels, on the grounds that educational-level is strongly associated with socioeconomic position [5]. Our data are consistent with this: 79% of participants aged 30-49 with a degree-level qualification in Natsal-3, and 74% in FECOND, were in managerial and professional positions. In the British survey, we assigned parents who had never had a job or who were partly skilled or unskilled to the lower socioeconomic group, those in technical and skilled positions to the middle group, and those in professional and managerial occupations to the higher group. In the French survey, we assigned parents who had no qualifications to the lower group, those with baccalaureate or less to the middle group, and those with a degree-level qualification to the higher group. As information on parent’s characteristics was collected differently in the two surveys, this variable measures relative, not absolute disadvantage. Non-response to the question on parent characteristics was relatively high – roughly ten percent – in both surveys. Examination of item non-responders showed that on other characteristics they more closely resembled respondents from lower socioeconomic backgrounds. In order to not lose a large number of respondents from the analysis, and not bias the results towards respondents from higher socioeconomic groups, we created a fourth ‘not answered’ category in this variable. For all other variables in this analysis missing data was less than 2% [6].

*Analysis*

We first described the two survey samples of men and women aged 17-29 years in terms of educational-level, parental socioeconomic group, and outcomes on the pathway to abortion, and described the differences between Britain and France and between men and women. Each analysis was restricted to respondents who had had the chance to experience the outcome of interest. Analyses of contraceptive-use at first sex were run on respondents who reported ever having had sex, and analyses of conception before aged 20 were run on respondents sexually experienced by age 20. Finally, analyses of conception before 20 and recourse to abortion among women who reported a conception before 20 were restricted to women aged older than 20 years and three months at interview. This was in order to include women who were pregnant at interview and was based on the assumption that women are likely to be aware of their pregnancy by three months gestation. We used bi-variate analysis to examine the relationships between reporting each of these outcomes and educational-level and parent relative socioeconomic group among men and women. Finally, we used multivariable logistic regression to examine these associations, adjusting for family structure at age 14/15 (whether the respondent lived with both natural parents at this age) as this was identified as a potential confounder. For all outcomes except reporting of sex before 16 we adjusted for age at first sex. In order to assess whether the strength of the association between socioeconomic characteristics and outcomes differed between men and women and between Britain and France, we tested for interactions of interactions of each of the two socioeconomic variables with sex and with country. All analyses were run on complete cases.

**References**

[1] Erens B, Phelps A, Clifton S, Mercer CH, Tanton C, Hussey D, et al. Methodology of the third British National Survey of Sexual Attitudes and Lifestyles (Natsal-3). Sexually Transmitted Infections 2014;90:84–9. doi:10.1136/sextrans-2013-051359.

[2] Legleye S, Charrance G, Razafindratsima N, Bohet a., Bajos N, Moreau C. Improving Survey Participation: Cost Effectiveness of Callbacks to Refusals and Increased Call Attempts in a National Telephone Survey in France. Public Opinion Quarterly 2013;77:666–95. doi:10.1093/poq/nft031.

[3] Jones RK, Kost K. Underreporting of induced and spontaneous abortion in the United States: An analysis of the 2002 National Survey of Family Growth. Studies in Family Planning 2007;38:187–97.

[4] Scott R. Understanding differences in conception and abortion rates among under 20s in Britain and France: Examining the role of disadvantage. PhD thesis: London School of Hygiene and Tropical Medicine, 2016.

[5] Krieger N, Williams DR, Moss NE. Measuring social class in US public health research: concepts, methodologies, and guidelines. Annual Review of Public Health 1997;18:341–78. doi:10.1146/annurev.publhealth.18.1.341.

[6] Mercer CH, Tanton C, Prah P, Erens B, Sonnenberg P, Clifton S, et al. Changes in sexual attitudes and lifestyles in Britain through the life course and over time: findings from the National Surveys of Sexual Attitudes and Lifestyles (Natsal). Lancet 2013;382:1781–94. doi:10.1016/S0140-6736(13)62035-8.
